# Evaluating Immunologic and Illness Outcomes of SARS-CoV-2 Infection in Vaccinated and Unvaccinated Children Aged ≥ 5 Years, in a Multisite Longitudinal Cohort

**DOI:** 10.3390/diseases12080171

**Published:** 2024-08-01

**Authors:** Cynthia Porter, Zoe L. Lyski, Jennifer L. Uhrlaub, Katherine D. Ellingson, Zuha Jeddy, Lisa Gwynn, Patrick Rivers, Ryan Sprissler, Kurt T. Hegmann, Melissa M. Coughlin, Ashley L. Fowlkes, James Hollister, Lindsay LeClair, Josephine Mak, Shawn C. Beitel, Sammantha Fuller, Pearl Q. Zheng, Molly Vaughan, Ramona P. Rai, Lauren Grant, Gabriella Newes-Adeyi, Young M. Yoo, Lauren Olsho, Jefferey L. Burgess, Alberto J. Caban-Martinez, Sarang K. Yoon, Amadea Britton, Manjusha Gaglani, Andrew L. Phillips, Matthew S. Thiese, Melissa Briggs Hagen, Jefferson M. Jones, Karen Lutrick

**Affiliations:** 1 Department of Epidemiology and Biostatistics, Mel and Enid Zuckerman College of Public Health, University of Arizona, Tucson, AZ 85724, USA; 2 Immunobiology, College of Medicine—Tucson, University of Arizona, Health Sciences, Tucson, AZ 85724, USA; 3 Abt Associates, Rockville, MD 20852, USA; 4 Leonard M. Miller School of Medicine, University of Miami, Miami, FL 33136, USA; 5 Family and Community Medicine, College of Medicine—Tucson, University of Arizona Health Sciences, Tucson, AZ 85711, USA; 6 Center for Applied Genetics and Genomic Medicine, University of Arizona, Tucson, AZ 85721, USA; 7 Rocky Mountain Center for Occupational and Environmental Health, Department of Family and Preventive Medicine, University of Utah Health, Salt Lake City, UT 84111, USA; 8 Coronavirus and Other Respiratory Viruses Division, National Center for Immunization and Respiratory Diseases, Centers for Disease Control and Prevention, Atlanta, GA 30333, USA; 9 Influenza Division, National Center for Immunization and Respiratory Diseases, Centers for Disease Control and Prevention, Atlanta, GA 30333, USA; 10 Baylor Scott & White Health, Texas A&M University College of Medicine, Temple, TX 76508, USA

**Keywords:** SARS-CoV-2, antibody response, vaccination, infection, children

## Abstract

**Simple Summary:**

Children with prior vaccine-induced immunity at the time of infection had more robust antibody binding against the receptor-binding domain (RBD) and S2 domain of Spike, and experienced fever less often compared to unvaccinated children during primary SARS-CoV-2 infection.

**Abstract:**

Hybrid immunity, as a result of infection and vaccination to SARS-CoV-2, has been well studied in adults but limited evidence is available in children. We evaluated the antibody responses to primary SARS-CoV-2 infection among vaccinated and unvaccinated children aged ≥ 5 years. Methods: A longitudinal cohort study of children aged ≥ 5 was conducted during August 2021–August 2022, at sites in Arizona, Texas, Utah, and Florida. Children submitted weekly nasal swabs for PCR testing and provided sera 14–59 days after PCR-confirmed SARS-CoV-2 infection. Antibodies were measured by ELISA against the receptor-binding domain (RBD) and S2 domain of ancestral Spike (WA1), in addition to Omicron (BA.2) RBD, following infection in children, with and without prior monovalent ancestral mRNA COVID-19 vaccination. Results: Among the 257 participants aged 5 to 18 years, 166 (65%) had received at least two mRNA COVID-19 vaccine doses ≥ 14 days prior to infection. Of these, 53 occurred during Delta predominance, with 37 (70%) unvaccinated at the time of infection. The remaining 204 infections occurred during Omicron predominance, with 53 (26%) participants unvaccinated. After adjusting for weight, age, symptomatic infection, and gender, significantly higher mean RBD AUC values were observed among the vaccinated group compared to the unvaccinated group for both WA1 and Omicron (*p* < 0.0001). A smaller percentage of vaccinated children reported fever during illness, with 55 (33%) reporting fever compared to 44 (48%) unvaccinated children reporting fever (*p* = 0.021). Conclusions: Children with vaccine-induced immunity at the time of SARS-CoV-2 infection had higher antibody levels during convalescence and experienced less fever compared to unvaccinated children during infection.

## 1. Background

The humoral immune response to infection with severe acute respiratory syndrome coronavirus 2 (SARS-CoV-2), the causative agent of coronavirus disease 2019 (COVID-19), has been extensively studied in adults [1,2,3,4]; however, information regarding the antibody response in children is more limited, but of high public health importance. Children are less likely to experience severe disease compared to adults and may have different humoral immune responses to infection [5,6,7].

Differences in neutralizing antibodies against SARS-CoV-2 infection have been observed in different age groups, with children younger than three years exhibiting the highest antibody titers [8,9,10] and children younger than 12 years exhibiting higher titers of binding and neutralizing SARS-CoV-2-specific antibodies compared to adolescents and adults following infection with an origin strain of SARS-CoV-2 (WA1) [1,8,11]. Following infection or vaccination with two doses of monovalent ancestral mRNA COVID-19 vaccine, elevated binding and neutralizing antibodies are present in adults and children; in both cases, SARS-CoV-2-specific antibodies remain detectable six months to one year post-infection or vaccination [9,12,13,14,15].

At the beginning of 2022, an increased incidence of SARS-CoV-2 infections was observed, largely due to the highly transmissible Omicron variant. During this time, children aged 5 years and older were eligible for COVID-19 vaccination in the United States [16]. This rise in infections resulted in a noticeable increase in hybrid immunity. In adults, hybrid immunity has been shown to result in superior antibody responses compared to infection or vaccination alone [17,18]. Specifically, adults with hybrid immunity exhibit higher antibody titers, increased antibody durability (greater than 7 months), greater neutralization against variants of concern, and a reduced risk of infection when compared to adults who are only infected or vaccinated [17,18]. However, studies are limited in children and adolescents [19,20,21].

The PROTECT (Pediatric Research Observing Trends and Exposures in COVID-19 Timelines) study provides an opportunity to assess antibody responses to primary SARS-CoV-2 infections [22]. Previous investigations using the PROTECT cohort demonstrated that children aged 5–11 years exhibit a robust antibody response following the primary monovalent ancestral mRNA COVID-19 vaccine series, and those with the highest magnitude antibody responses were less likely to experience post-vaccine infections [23]. The aim of the present investigation is to evaluate SARS-CoV-2-specific antibody responses following in-study, SARS-CoV-2 infection, confirmed by real-time reverse transcription polymerase chain reaction (rRT-PCR). Children in this analysis were either unvaccinated or had received two or three doses of ancestral monovalent mRNA COVID-19 vaccination 14 or more days prior to infection. This study aims to add to the body of knowledge regarding the antibody response and illness symptoms after initial SARS-CoV-2 infection among pediatric groups who were unvaccinated or vaccinated at the time of infection.

## 2. Methods

### 2.1. Study Design

The PROTECT study, enrolling children aged 6 months to 17 years, was initiated in July 2021 in four states: Arizona, Florida, Texas, and Utah; the protocols have been previously described [22]. PROTECT is an ancillary study by the Arizona Healthcare, Emergency Response, and Other Essential Workers Study and Research on the Epidemiology of SARS-CoV-2 in Essential Response Personnel (HEROES-RECOVER) network, which is composed of two large prospective cohorts of adult participants [24,25].

Children of HEROES-RECOVER participants and from other community members were recruited for the study. Parents/legal guardians provided informed consent and children aged 12 to 17 years provided assent for study participation. The study protocol was approved by the Abt Associates Institutional Review Board (IRB) (which serves as the single IRB of record for the Florida, Texas, and Utah sites and Centers for Disease Control and Prevention [CDC]) and by the University of Arizona IRB (for the Arizona site).

### 2.2. Inclusion and Exclusion Criteria

Children were included in the analysis if (1) they experienced a primary SARS-CoV-2 infection during the study period, namely August 2021–August 2022, determined by the earliest available post-infection blood draws, (2) they were five years of age or older (due to the timing of vaccine eligibility), (3) they had no self-reported history of SARS-CoV-2 infection, (4) they were either unvaccinated or fully vaccinated (received two or three doses of ancestral monovalent Pfizer-BioNTech COVID-19 mRNA vaccine, Moderna COVID-19 mRNA-1273 or a combination of the two vaccines 14 days or more prior to infection, and (5) they provided an in-study SARS-CoV-2 post-infection blood draw during the study period, and (6) within 14–59 days of infection. Baseline antibody test results were not used in the primary analysis because blood draws were optional and not completed by all participants. Children were excluded if they had only one dose of monovalent COVID-19 mRNA vaccine or obtained an additional vaccine dose after infection but prior to specimen collection or had reported a prior infection at the time of enrollment.

### 2.3. Data Collection

Upon enrollment, children or their parents/legal guardians provided sociodemographic information, school/daycare attendance, percent of time wearing masks outside the home, COVID-19 infection history, health insurance coverage, and medical history (e.g., influenza and other childhood vaccines, medical conditions, and daily medication use). The COVID-19 vaccination status was self- or parent-reported through electronic surveys and was verified primarily by vaccination cards, electronic medical records, or state Immunization Information Systems. Children provided a self-collected or caregiver-collected/assisted weekly mid-turbinate nasal specimen for SARS-CoV-2 testing at the Marshfield Clinical Research Laboratories (Marshfield, WI) and provided self-reported information regarding COVID-19 symptoms each week. Participants were asked to provide an additional specimen at symptom onset [22]. The predominant circulating SARS-CoV-2 variant was determined when the national prevalence exceeded 50% of the current circulating viral variants [26]. The study period coincided with the Delta (August 2021–December 2021) and Omicron (December 2021–August 2022) periods, with most infections occurring from Omicron [27].

### 2.4. Blood Collection

Blood collection was not required for inclusion in the PROTECT study. For this investigation, participating children could provide a blood specimen at enrollment and again 14–59 days following the in-study primary SARS-CoV-2 infection. Serum samples were obtained from 5–10 mL of whole blood, then frozen and transported on dry ice to the University of Arizona, where they were stored at −80 °C until tested [28].

### 2.5. Enzyme-Linked Immunosorbent Assay (EL–ISA)

Sera were sent to the University of Arizona Genetics Core laboratory for serologic testing. ELISAs were used to measure the binding antibodies specific to SARS-CoV-2 WA1 and Omicron RBD, as well as WA1 S2, as previously described [29]. Briefly, five 3-fold dilutions of sera were made starting at a 1:60 dilution and reported as the endpoint titer. The optical density was measured, and the antibody levels were reported as areas under the curve (AUC) [29,30,31]. Qualitative ELISAs against RBD and S2 were conducted on serum collected at enrollment (if provided) in unvaccinated children to verify no prior SARS-CoV-2 infection. These were not used to determine the prior infection status in this analysis for children who were already vaccinated at the time of enrollment [32].

### 2.6. Statistical Methods

The antibody response to infection was assessed by calculating the antibody AUC for RBD and S2 antibodies. Descriptive statistics of the participant demographics were generated and used to examine differences in the humoral immune response by vaccination status at infection. Differences between groups were separately assessed using a Kruskal–Wallis test, where *p* values < 0.05 were considered statistically significant. Variables were assessed for collinearity.

To further examine the relationship between the post-infection antibody response and previous vaccination status, multiple linear regression was used to examine the AUC values, adjusting for the age category, body mass index (BMI) percentile for age, gender, and symptom status (dichotomized as asymptomatic or any symptoms) [6,7,8]. Because the AUC values were not normally distributed, they were log transformed. Separate models were generated for the WA1 and BA.2 RBD antibody results. The proportional odds assumption and the goodness of fit were examined through the Hosmer and Lemeshow test. The results of the ordinal models and descriptive tables with time to vaccination are listed in the Supplemental Materials (Tables S1 and S2). All analyses were conducted in SAS v9.4 (SAS Inc., Cary, NC, USA) and Graphpad Prism (version 9.4.1 for Mac, GraphPad Software, San Diego, CA, USA).

### 2.7. Sensitivity Analysis

To test the robustness of the models, we used Firth’s method for the bias correction of the proportional odds regression estimator, previously used by Lipitz et al. [33]. A second sensitivity analysis was conducted by assessing participants that potentially had missed SARS-CoV-2 infections at enrollment due to limitations in the laboratory methods mentioned previously, removing participants who were unvaccinated at the time of enrollment who had a positive qualitative ELISA result measured by RBD or S2. This was to assess only those who had self-reported results and were unable to confirm their previous infection status through a blood draw.

## 3. Results

**Figure 1:**
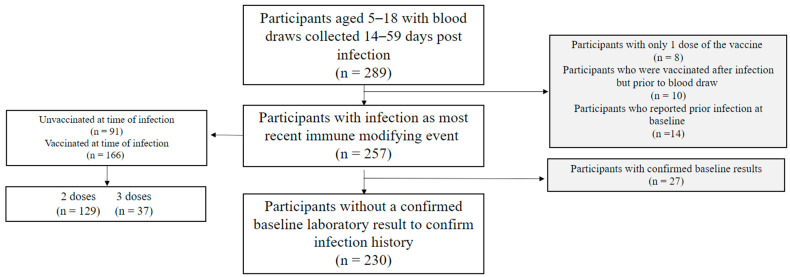
Participant flow diagram for children in the PROTECT study (August 2021–August 2022).

From August 2021 to August 2022, 289 children provided a blood specimen 14–59 days post-infection (Figure 1). Children who had obtained only one dose of the vaccine (n = 8), were vaccinated after infection but prior to blood draw (n = 10), or had reported a previous infection at baseline (n = 14) were excluded, bringing the total sample size to 257.

Of the 257 children, 166 (65%) were vaccinated at the time of infection (Table 1). Of those, 129 (78%) received two doses and 37 (22%) received three doses of ancestral monovalent mRNA COVID-19 vaccine, either Pfizer-BioNTech or a combination of the Moderna mRNA-1273 and Pfizer-BioNTech vaccines. In addition, 204 (79%) had infections that coincided with Omicron variant predominance, while 53 (21%) experienced an infection during Delta predominance.

Of the analytic sample, 123 (48%) were female, 165 (64%) were non-Hispanic white, and 64 (25%) were Hispanic; 31 (12%) were overweight or obese; 221 (86%) did not have chronic conditions; and 204 (79%) were from study sites in Arizona, 13 (5%) were from Temple, TX, 11 (4%) were from Miami, FL, and 29 (11%) were from Salt Lake City, UT.

In addition, 212 (82%) reported symptomatic infection and had PCR-positive results lasting an average of 1.5 weeks (standard deviation [SD] 1.3). Children spent a mean of 1.7 (SD 1.8) days in bed during their illnesses, and 99 (38%) reported fever and experienced symptoms lasting an average of 8.6 (SD 7.9) days. Symptoms during illness did not vary significantly for vaccinated or unvaccinated children. Significant differences in illness characteristics were observed in the duration of PCR positivity, with a shorter average number of weeks (1.2, SD 1.0, vs. 1.6 SD 1.4, *p* = 0.028) and a higher incidence of fever (48%, vs. 33%, *p* = 0.021) in unvaccinated participants compared to vaccinated participants. Significant differences were also observed between the site and vaccination status (*p* = 0.020) (Table 1).

### 3.1. Association of Immune Response and Vaccination Status

Our unadjusted linear regression models examined the relationship between vaccination status and the WA1 RBD antibody response and found that the post-infection antibody AUC values were 340% higher in vaccinated children compared to unvaccinated children (geometric mean ratio [GMR]: 4.4 (95% CI: 3.8, 5.1) *p* ≤ 0.0001) (Supplemental Table S1). After adjusting for weight, age, symptomatic infection, and gender, there remained 330% higher WA1 RBD antibody AUC values among vaccinated children compared to unvaccinated children (GMR: 4.3 (95% CI: 3.8, 5.0) *p* ≤ 0.0001). After adjustment in the BA.2 models, there were 260% higher BA.2 RBD antibody AUC values among vaccinated compared to unvaccinated children (GMR: 3.6 (95% CI: 3.2, 4.1) *p* ≤ 0.0001). There was no change in the GMR between the unadjusted and adjusted BA.2 models. These findings agree with the adjusted and unadjusted ordinal logistic regression models examining WA1 and BA.2 RBD antibodies using end titers (Supplemental Table S2).

After removing unvaccinated children with positive baseline qualitative ELISA results (n = 27) in the adjusted models, 340% higher WA1 RBD antibody AUC values were observed post-infection among vaccinated compared with unvaccinated participants (GMR: 4.4, 95% CI: (3.8, 5.1) *p* ≤ 0.0001) and 260% higher BA.2 RBD antibody AUC values were observed compared with unvaccinated participants (GMR: 3.6, 95% CI: (3.2, 4.2) *p* ≤ 0.0001) (Supplemental Table S1). These findings agree with the unadjusted and adjusted analysis.

**Figure 2:**
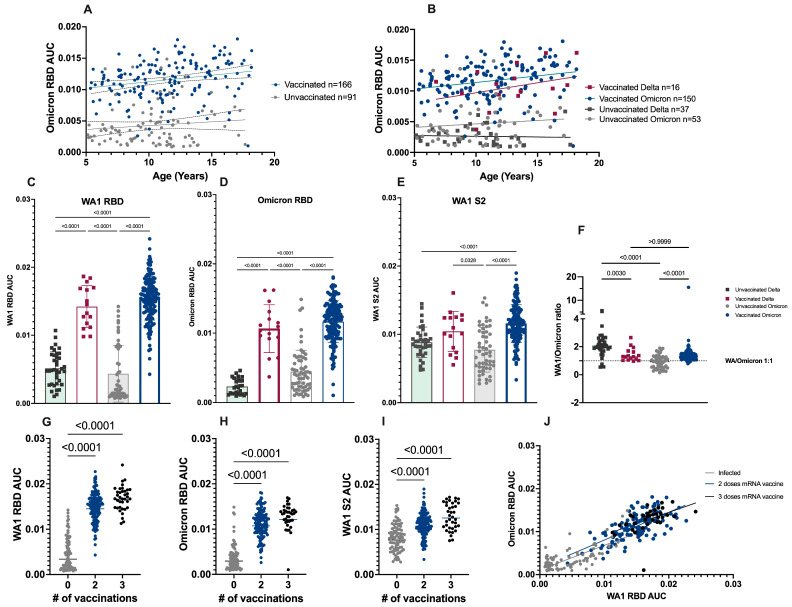
The vaccination status and number of monovalent mRNA doses influence the magnitude of antibody responses following primary SARS-CoV-2 infection. A–B Slopes were determined by simple linear regression. (**A**) Omicron RBD antibody AUC by age at the time of infection. The slopes of the lines (indicating a positive or negative relationship with age) are not significantly different *p* = 0.503. (**B**) Omicron RBD antibody AUC values further stratified by variant. The slopes of the lines are not significant (*p* = 0.469); however, antibody AUC values are significantly different by vaccination status (*p* < 0.0001). (**C**–**F**) *p* = values are based on non-parametric one-way ANOVA, *p* < 0.0001 for all antigens tested (WA1 RBD, BA.2 RBD, and WA1 S2). (**F**) Ratio of WA1/BA.2 AUC values for each individual across groups. The dashed line at 1 indicates a 1:1 relationship of antibody binding to ancestral and variant RBD. (**G**–**J**) Significant differences observed in all groups between zero and two or three doses (*p* < 0.0001) by non-parametric one-way ANOVA. No significant difference was observed between two and three doses in any group (*p* = 0.135, *p* = 0.121, *p* = 0.059). (**J**) The relationship between BA.2 RBD and WA1 RBD by the number of vaccinations (grey= unvaccinated, blue = 2 doses, black = 3 doses) at the time of infection. The slopes are not significantly different *p* = 0.78.

Statistically significant differences were not observed between age groups and the antibody AUC (*p* = 0.05); however, statistically significant differences in the mean antibody AUC values for both WA1 and BA.2 RBD antibodies (*p* < 0.0001) were observed when stratified by vaccination status, with higher values among the previously vaccinated group (Figure 2A), regardless of whether infection was with Delta or Omicron (Figure 2B), or what antigen was tested (Figure 2C–E). The WA1 RBD mean antibody AUC values (Figure 2C) were four times higher among vaccinated compared to unvaccinated children (0.003 vs. 0.01, *p* < 0.0001). A similar trend was observed for BA.2 RBD antibodies (Figure 2D), where the mean AUC in vaccinated children was 3.3 times that observed in unvaccinated children. The smallest differences in the antibody response between vaccinated and unvaccinated children were observed for WA1 S2-binding antibodies, where the mean S2 antibodies were 1.4 times higher in vaccinated compared to unvaccinated children (Figure 2E).

### 3.2. Ratio of WA1 to Omicron Binding Antibodies

The ratio of WA1 to BA.2 RBD-binding antibodies was further explored (Figure 2F) by plotting the ratio of the WA1 RBD antibody AUC values over the BA.2 RBD antibody AUC values (for each child individually). Among unvaccinated children, there was a significant difference in the mean WA1/BA.2 ratios (*p* < 0.0001) by variant, with a GMR of 2.0 for Delta-infected participants and 0.8 for BA.2-infected participants. In vaccinated children, there was no significant difference in the mean WA1/BA.2 ratios (GMR for both: 1.4).

### 3.3. Antibody Response to Primary Infection by Number of COVID-19 Vaccine Doses

The post-infection RBD antibody AUC values were stratified by the number of ancestral monovalent mRNA COVID-19 vaccines received (Figure 2G–J). The RBD antibody AUC values increased with additional vaccine doses for all antigens tested. A significant difference was observed between zero and either two or three doses of ancestral monovalent mRNA COVID-19 vaccine (*p* < 0.0001), as noted above. However, there was no significant difference between two or three doses of vaccine (*p* < 0.0001). The relationship between the BA.2 and WA1 RBD antibody AUC values was explored (Figure 2J) to determine whether skewing towards antibody-binding WA1 or BA.2 was occurring based on the number of vaccine doses. The slopes of the regression lines were not significantly different (*p* = 0.78), indicating that the relationship between BA.2 and WA1-binding antibodies does not change with additional ancestral vaccine doses; however, there was a trend towards higher AUC values (both WA1 RBD and BA.2 RBD) with a greater number of vaccine doses.

## 4. Discussion

Here, we examined the antibody responses among children aged ≥5 years with and without prior ancestral monovalent mRNA COVID-19 vaccination at the time of infection. A relationship between the antibody response and age was not observed; however, vaccinated children who received at least two doses of ancestral monovalent mRNA COVID-19 vaccine prior to infection exhibited antibody responses of higher magnitude compared to those who were unvaccinated at the time of infection, for both RBD (WA1 and BA.2) and S2-binding antibodies (WA1) irrespective of the infecting virus (Delta or Omicron). These increased antibody titers (up to four times higher in vaccinated compared to unvaccinated against WA1 RBD) can translate to lower odds of experiencing subsequent SARS-CoV-2 infections, as previously shown by our group [23]. In this study, no significant differences in the mean antibody AUC values were observed between two or three doses of monovalent mRNA vaccination; for RBD (WA1 and BA.2) and S2 (WA1), however, differences in antibody affinity, the neutralization breadth, and durability are expected to differ greatly with additional antigen exposures [17,21,34].

Previous research has found that vaccination in children protects against multisystem inflammatory syndrome and death; consistent with that, no cases of severe illness were reported in this study [2,35,36]. Here, it was observed that children vaccinated prior to infection experienced a longer mean duration of PCR positivity, as measured by weekly PCR testing, but were symptomatic for fewer days, with a smaller proportion experiencing fever compared to unvaccinated children. This is an important finding and further supports the importance of vaccination in limiting disease symptoms.

This study has multiple limitations. First, it is possible that previously infected participants did not report infection prior to enrollment. Blood draws at enrollment were not mandatory, resulting in limited baseline serological data. However, our sensitivity analysis suggests that this did not greatly influence the study findings. Secondly, using PCR positivity to determine illness duration may not provide information about differences in the duration of infectivity among vaccinated or unvaccinated groups, because PCR can detect nucleic acids from viruses that are no longer infectious [37,38,39,40,41]. Third, children less than 5 years of age were not eligible for vaccination during this period and not included in this analysis. Fourth, these results are not generalizable to updated vaccine formulations (e.g., bivalent or XBB.1.5 monovalent mRNA vaccine). Fifth, none of the children in this study received a primary series of Moderna mRNA-1273. Sixth, the convalescent blood draws were performed 14–59 days post-infection for all children; however, the time between immune-modifying events (i.e., vaccination and infection) was not included in the analysis. Children in this cohort experienced mild symptoms during infection and did not have many comorbid conditions. Despite significant differences between the site and vaccination status, the study site was not able to be included in the model due to a lack of model convergence and small enrollment numbers at some sites. However, statistically significant differences were not observed between the antibody response and site.

The strengths of this study are that it includes a large prospective cohort of school-age children and adolescents, allowing for symptomatic and asymptomatic SARS-CoV-2 infections to be captured in real-time due to weekly PCR testing. Second, blood draws were collected 14–59 days post-infection, allowing for controlled comparisons between groups. Third, the antibody responses to both WA1 and Omicron were examined to compare the immune response across different variants.

## 5. Conclusions

In conclusion, children who were vaccinated at the time of infection had higher antibody levels during convalescence compared to unvaccinated children, and lower proportions reported fever during SARS-CoV-2 infection. More research is needed to understand the impact of varying numbers of antigen exposures, timing of exposure, order of immune-modifying events, and comorbid conditions, as well as differences by vaccination status with the bivalent and monovalent XBB.1.5 boosters.

## Figures and Tables

**Table 1:** Participant characteristics among children and adolescents with first-time SARS-CoV-2 infections by vaccination status among the PROTECT cohort, August 2021 to August 2022, (n = 257).

Participant Characteristics n, %	Unvaccinated n (%) (Total = 91)	Vaccinated ^a^ n (%) (Total = 166)	Total (n = 157)	*p*-Value ^b^
Variant				<0.001 *
Delta	37 (40.7)	16 (9.6)	53 (20.6)	
Omicron	54 (59.3)	150 (90.4)	204 (79.4)	
Gender ^b^				0.071
Female	36 (39.6)	87 (52.4)	123 (47.9)	
Male	54 (59.3)	79 (47.6)	133 (51.8)	
Other Gender Identity	1 (1.1)	0 (0.0)	1 (0.3)	
Study Site ^b^				0.020 *
Temple, TX	7 (7.7)	6 (3.6)	13 (5.1)	
Tucson, AZ	36 (39.6)	91 (54.8)	127 (49.4)	
Salt Lake City, UT	15 (16.5)	14 (8.4)	29 (11.3)	
Miami, FL	7 (7.7)	4 (2.4)	11 (4.3)	
Phoenix, AZ	16 (17.6)	38 (22.9)	54 (21.0)	
Other places in AZ	10 (10.9)	13 (7.8)	23 (8.9)	
Race/Ethnicity ^b^				0.229
NH White	60 (66.7)	105 (63.3)	165 (64.2)	
NH Black	0 (0.0)	4 (2.4)	4 (1.6)	
NH Asian	3 (3.0)	1 (0.6)	4 (1.6)	
Hispanic	21 (21.8)	42 (25.3)	64 (24.9)	
Not listed/Refused	7 (8.5)	14 (8.4)	21 (7.7)	
Weight Status ^b,c^				0.696
Not overweight or obese	81 (89.0)	145 (87.4)	226 (87.9)	
Overweight or obese	10 (11.0)	21 (12.7)	31 (12.1)	
Symptom Status ^b^				0.982
Asymptomatic	16 (17.6)	29 (17.5)	45 (17.5)	
Symptomatic	75 (82.4)	137 (82.5)	212 (82.5)	
Weeks of PCR positivity ^d^ mean, SD	1.2 (1.0)	1.6 (1.4)	1.5 (1.3)	0.028 *
Length of symptoms in days mean, SD	9.2 (8.4)	8.2 (7.7)	8.6 (7.9)	0.382
Days spent in bed, mean, SD	1.7 (1.8)	1.7 (1.8)	1.7 (1.8)	0.785
Fever ^e^	44 (47.8)	55 (33.1)	99 (38.5)	0.021 *
Days from illness to blood draw mean, SD	34.0 (9.9)	33.3 (9.0)	33.5 (9.3)	0.605
Comorbid Conditions ^f^				0.779
0	79 (86.8)	142 (85.5)	221 (86.0)	
1+	12 (13.2)	24 (14.5)	36 (14.0)	

* Abbreviations: SD—Standard Deviation PCR—Polymerase Chain Reaction. a: Vaccinated defined as 2 or more doses of the monovalent COVID-19 mRNA vaccine. b: *p* value comes from chi square test for statistical significance or Fisher’s exact test between vaccination groups. c: Overweight categorized as BMI for sex and age equal to or greater than the 85th percentile, but less than the 95th percentile. d: PCR positivity calculated as the difference from the first day of PCR positivity to the last day of PCR positivity, divided by 7. e: Above average body temperature (higher than 98.6 degrees F). f: Chronic conditions included: asthma, chronic lung disease, cancer, diabetes, heart disease, hypertension, immunosuppression, kidney disease, liver disease, neurologic or neuromuscular disease or disorder, and autoimmune disease.

## Data Availability

Study data are not publicly available.

## Supplementary Materials

The following supporting information can be downloaded at: https://www.mdpi.com/article/10.3390/diseases12080171/s1, Table S1: Unadjusted and adjusted multiple linear regression models examining the association between immune response and vaccination status at the time of infection among children aged 5–17 years in the PROTECT cohort, August 2021 to August 2022, (n = 257); Table S2: Unadjusted and adjusted ordinal logistic regression models examining the association between immune response (at 3-fold increments) and vaccination at the time of infection among children aged 5–17 years in the PROTECT cohort (n = 257); Table S3: Mean AUC values by days from vaccination to infection among PROTECT participants (n = 166); Table S4: Days from vaccination to infection among vaccinated PROTECT participants (n = 166).

## Abbreviations

AUC	Area under the curve
BA.2	Omicron
BMI	Body Mass Index
CDC	Centers for Disease Control and Prevention
COVID-19	Coronavirus disease 2019
ELISA	Enzyme-linked immunosorbent assays
GMR	Geometric mean ratio
IRB	Institutional review board
OR	Odds Ratio
RBD	Receptor binding domain
rRT-PCR	Real-time reverse transcription polymerase chain reaction
SARS-CoV-2	severe acute respiratory syndrome coronavirus 2
S2	S2 region of spike protein
PROTECT	Pediatric Research Observing Trends and Exposures in COVID-19 Timelines
HEROES-RECOVER	Arizona Healthcare, Emergency Response, and Other Essential Workers Study and Research on the Epidemiology of SARS-CoV-2 in Essential Response Personnel
WA1	Washington-1
95% CI	95% confidence interval
