# Supplementary material for: Evaluating Immunologic and Illness Outcomes of SARS-CoV-2 Infection in Vaccinated and Unvaccinated Children Aged ≥ 5 Years, in a Multisite Longitudinal Cohort
Source: Diseases. 2024 Aug 1;12(8):171. doi: 10.3390/diseases12080171 (PMC11354143; doi:10.3390/diseases12080171)
Supplement: Supplementary file 1 [file diseases-12-00171-s001.zip › diseases-3098186-supplementary.pdf]

Appendix A: Supplementary Tables

Supplemental Table 1: Unadjusted and adjusted multiple linear regression models examining the association between immune response and vaccination status at the time of infection among children aged 5-17 years in the PROTECT cohort, August 2021 to August 2022, (n=257).

	Unadjusted (n=257)		Adjusted [a] (n=257)		Sensitivity #2[a,b] (n=230)	
	Geometric Mean Ratio (95% CI)	P value	Geometric Mean Ratio (95% CI)	P value	Geometric Mean Ratio (95% CI)	P value
WA1 RBD[c]						
Unvaccinated	Ref		Ref		Ref	
Vaccinated[d]	4.4 (3.8, 5.1)	<0.0001	4.3 (3.8, 5.0)	<0.0001	4.4 (3.8, 5.1)	<0.0001
BA.2 RBD[e]						
Unvaccinated	Ref		Ref		Ref	
Vaccinated[d]	3.6 (3.2, 4.1)	<0.0001	3.6 (3.2, 4.1)	<0.0001	3.6 (3.2, 4.2)	<0.0001

*Abbreviations: CI- Confidence Interval

a: Adjusted for overweight/obese status, age category, symptomatic infection, and gender (collapsed into two categories to account for sparse cells)

b: Excluding unvaccinated participants with positive baseline qualitative results

c: WA1 RBD antibody area under the curve (AUC) value

d: Vaccinated defined as 2 or more doses of the monovalent COVID-19 mRNA vaccine

Supplemental table 2: Unadjusted and adjusted ordinal logistic regression models examining the association between immune response (at 3-fold increments) and vaccination at the time of infection among children aged 5-17 years in the PROTECT cohort (n=257)

	Unadjusted (n=257)		Adjusted[a] (n=257)		Sensitivity #1[a,b] (n=257)		Sensitivity #2[a,b,c] (n=230)	
	OR (95% CI)	P value	OR (95% CI)	P value	OR (95% CI)	P value	OR (95% CI)	P value

WA1 RBD[d]								
Unvaccinated	Ref		Ref		Ref		Ref	
Vaccinated[e]	173.7 (68.2, 442.15)	<0.0001	188.7 (73.3, 485.9)	<0.0001	157.6 (63.6, 390.4)	<0.0001	253.3 (86.4, 742.6)	<0.0001
BA.2 RBD[f]								
Unvaccinated	Ref		Ref		Ref		Ref	
Vaccinated[e]	39.2 (20.0, 76.6)	<0.0001	36.9 (18.9, 71.9)	<0.0001	34.0 (17.5, 66.0)	<0.0001	37.7 (18.6, 76.1)	<0.0001

*Abbreviation: CI – confidence interval

a: Adjusted for overweight status, age category, symptomatic infection, and gender (collapsed into two categories to account for sparse cells)

b: Using Firth's method for bias correction

c: Excluding unvaccinated participants with positive baseline qualitative results

d: WA1 RBD antibody area under the curve (AUC) value

e: Vaccinated defined as 2 or more doses of the monovalent COVID-19 mRNA vaccine

Supplemental table 3: Mean AUC values by days from vaccination to infection among PROTECT participants (n=166)

Days from vaccination to infection	WA1 RBD AUC mean (SD)	BA.2 RBD AUC mean (SD)
**<150 days**	0.015 (0.003)	0.011 (0.003)
**150+ days**	0.016 (0.003)	0.012 (0.003)

Supplemental table 4: Days from vaccination to infection among vaccinated PROTECT participants (n=166)

**Days from Vaccination to Infection[a]**	**Mean (SD)**	**Minimum, Maximum**
Booster Dose	89.4 (67.9)	3, 183
Primary Series	135.5 (102.5)	20, 426

a: Considered fully vaccinated at 14 days from receipt of 2nd dose of the primary series.
